# Major Limitations of Cardiovascular Risk Scores

**DOI:** 10.1155/2024/4133365

**Published:** 2024-02-28

**Authors:** Ibtissam Talha, Noureddine Elkhoudri, Abderraouf Hilali

**Affiliations:** Laboratory of Health Sciences and Technologies, Higher Institute of Health Sciences of Settat, Hassan First University of Settat, Settat, Morocco

## Abstract

*Background*. Epidemiological studies conducted in extensive population cohorts have led to the creation of numerous cardiovascular risk predictor models. However, these tools have certain limitations that restrict its applicability. The aim behind the following work is to summarize today's best-known limitations of cardiovascular risk assessment models through presenting the critical analyses conducted in this area, with the intention of offering practitioners a comprehensive understanding of these restrictions. Critical analyses revealed that these scales exhibit numerous limitations that could impact their performance. Most of these models evaluate cardiovascular risk based on classic risk factors and other restrictions, thereby negatively affecting their sensitivity. Scientists have made significant advancements in improving cardiovascular risk models, tailoring them to accommodate a wide range of populations and devising scales for estimating cardiovascular risks that can account for all prevailing restrictions. Better understanding these limitations could improve the cardiovascular risk stratification.

## 1. Introduction

Cardiovascular disease stands as the foremost cause of mortality globally. In 2015, a staggering 17.7 million deaths resulted from cardiovascular diseases, representing 31% of global mortality [1]. Projections indicate an inexorable rise in these figures. By 2030, it is anticipated that cardiovascular diseases will claim over 23.6 million lives [2, 3].

Preventing cardiovascular disease is plausible. Strategies for primary prevention, classifying individuals based on an array of cardiovascular risk factors, have been proven highly effective [4]. While past approaches viewed cardiovascular risk factors individually, the current recommendation is a quantitative assessment of individuals' overall cardiovascular risk.

Assessing cardiovascular risk enables a comprehensive overview of cardiovascular health status. It serves a dual purpose: reassuring individuals with low cardiovascular risk and motivating them to uphold their cardiovascular health while also facilitating appropriate medical interventions for individuals with high cardiovascular risk [5, 6].

Epidemiological studies conducted in extensive population cohorts have led to the creation of numerous cardiovascular risk assessment tools. In 2011, the “Agency for Healthcare Research and Quality” identified 102 risk patterns, but only a handful underwent external validation. This validation is pivotal in appraising the performance and applicability of a risk model [6, 7]. The most commonly used cardiovascular risk models are the American and European scores [2]. These models differ in form, characteristics, and limitations. Hence, establishing a comprehensive understanding of the limitations of the most renowned cardiovascular risk scores to date is crucial for contributing to their enhancement. Existing literature in this domain has primarily focused on comparative studies between a few scores, often referencing the pioneering model, Framingham Heart Study (FHS). This article is aimed at presenting the current limitations of the best-known cardiovascular risk assessment models with a view to proposing recommendations for the improvement of these tools.

This investigation has identified 18 existing cardiovascular risk estimation models shown in Table 1: the Framingham Heart Study (FHS) [8–11], the Prospective Cardiovascular Münster Study (PROCAM) [12, 13] in two versions, ASSIGN [14], Systematic Coronary Risk Estimation (SCORE) in two versions [15], two versions of the Reynolds Risk Score (RRS) [16, 17], QRISK in two versions [18, 19], Girona del Cor Registry (REGICOR) [20], Dubbo study of the elderly [21], Atherosclerosis Risk in Communities (ARIC) Study [22], Progetto CUORE [23], WHO/ISH charts [24], and pooled cohort equation ASCVD [25].

Most of these tools predict the likelihood of a cardiovascular incident over a 10-year period, except for Framingham-30, a predictor over a 30-year span. Common risk factors across all models include age, sex, and smoking. Among the identified scores, several are derived from significant studies such as the Framingham Heart Study, many of which have undergone multiple modifications over the years. However, their use lacks standardization due to variances in population characteristics and performance variability [26, 27]. Cardiovascular risk scales typically present limitations in their temporal and spatial applicability. Based on continuous longitudinal surveys spanning several years, these models do not account for changes in cardiovascular morbidity and mortality [28]. The most effective cardiovascular risk model is one that offers the best comprehensive tools [29]. These models also exhibit geographical limitations, impacting their universal usefulness and applicability [30].

## 2. Major limitations

### 2.1. External Validation

In the realm of cardiovascular medicine, diverse methodologies exist for assessing cardiovascular risk. Tools like the Framingham risk engine, SCORE, and QRISK and other cardiovascular risk scores are employed. However, each calculator demonstrates distinct advantages for one population while potentially encountering limitations with another. Essentially, prediction tools excel for the population they were specifically studied and validated for.

Validating a risk prediction model externally is of utmost importance, as it furnishes essential evidence for evaluating its performance in a contemporary population and gauging its practical applicability. Comparative studies on the performance of cardiovascular risk prediction models often lack data and possess limitations, making generalized conclusions challenging [31]. Cooney et al. outlined three essential criteria for evaluating the performance of a cardiovascular risk model: discrimination, recalibration, and reclassification [32]. However, despite the precision in cardiovascular risk prediction, it remains a probability rather than a definitive value, offering an individual's average risk more than individual risk [33]. Recent studies on predictive models for cardiovascular disease (CVD) risk in the general population uncovered that a considerable number of these models have not undergone an external validation [34].

### 2.2. Cardiovascular History

The primary cardiovascular risk factors considered in assessing cardiovascular risk (CVR) do not include the CVD family history. For instance, the FHS serves as the foundation for the assessment models embedded within it and was the first study to confirm the relationship between Framingham risk factors (FRF) and cardiovascular disease. Nonetheless, it has limitations as it applies only to subjects without a cardiovascular history and does not incorporate family history as a cardiovascular risk factor [33]. In 2013, the American College of Cardiology (ACC) and the AHA adopted the pooled cohort equations, integrating data from the Framingham study cohort has rendered the Framingham risk score obsolete. As a result, the current use of the FHS is not recommended [35].

In Italy, the CUORE risk score was developed to provide a more precise representation of the 10-year cardiovascular disease (CVD) risk. However, the limited external validation of the CUORE risk score restricts its applicability in the Italian population. In Scotland, the ASSIGN score has undergone validation, demonstrating a slight superiority over Framingham and QRISK. However, it lacks external validation in other populations, cautioning against its use beyond the Scottish context. Likewise, the PROCAM risk score has not been validated in populations outside of Germany. Consequently, its application beyond Germany is not advisable [36].

### 2.3. Age

Most cardiovascular risk models underestimate risk in youth and overestimate it in the elderly, as these models primarily originated from cohorts where very young or elderly populations (over 75 years old) were in the minority [37, 38] and necessitate recalibration and validation for application across diverse populations. For instance, European guidelines advocate using the Systematic Coronary Risk Estimation (SCORE) to assess overall cardiovascular risk in the European population [15]. Nevertheless, it tended to overstate the risk of cardiovascular mortality in individuals aged 65–69 years and in those with normal blood pressure while concurrently underestimating the risk in hypertensive patients [35]. Additionally, SCORE does not acknowledge the observed decline in cardiovascular mortality and morbidity rates in Europe, relying on data collected over two decades ago [32]. Applying the SCORE table to an Australian-origin cohort predicted 666 cardiovascular deaths at 10 years, whereas observed deaths did not surpass 485 [39]. Subsequent studies found that the pooled cohort equation score tends to overstate cardiovascular risk, especially in older age groups [40].

### 2.4. Risk Factors

Common limitations across all cardiovascular risk scales include the imprecision of clinical measurements of certain risk factors, such as blood pressure and cholesterol. This imprecision severely impacts the identification of thresholds for incorporating therapeutic measures [41].

An overarching limitation common to all cardiovascular risk scales is their failure to account for the variable effects of cardiovascular risk factors across different age groups. The QRISK model attempted to address this by incorporating interaction variables between age and various CVRFs, yet this method lacks validation [35, 42].

### 2.5. Gender

Biologic, hormonal, and physiologic disparities between men and women lead to differences in CVD incidence. In women, CVR is at least 50% implying that gender stratification is essential to retrain the differences in CVR assessment tools [43]. Baart et al. identified 285 prediction models that have been developed for women in the general population in which only 9 were externally validated (Framingham with their five versions, SCORE, pooled cohort equations, and QRISK) and only two of them (1.3%) include female-specific predictors [44].

The FRS and the PROCAM risk algorithm are known to underestimate CHD risk in women. The Reynolds Risk Score (RRS) which was initially developed specifically for women tended to overestimate the CVR in a study on women's health [16]. Therefore, enhancing the current models could involve incorporating predictors specifically tailored for females as use of hormones, menopause and early menarche, pregnancy complications, primary ovarian insufficiency, and polycystic ovary syndrome [44].

### 2.6. New Risk Factors

In addition to the usual risk factors, a significant restriction lies in certain cardiovascular risk markers termed “new risk factors,” such as C-reactive protein, coronary calcium, and interleukin-6. These factors are not incorporated into cardiovascular risk models, though recent studies have shown their minor effect on adjusting the discrimination statistics of these scales [45, 46]. Studies have found a strong correlation between the level of ultrasensitive CRP and the occurrence of acute coronary syndromes and cardiovascular deaths [47, 48]. Furthermore, microalbuminuria indicates the onset of diabetic nephropathy, especially in type 1 diabetes (T1D) and poses a risk marker in diabetes mellitus (DM) when exceeding 30 mg per 24 hours. Hyperhomocysteinemia is also considered an independent risk factor for atherosclerosis by some researchers [49]. The new pooled cohort equations do not include “novel” risk markers that some consider important for risk assessment. However, the additional information provided by such markers has repeatedly been shown to be small, and their addition is typically only useful in intermediate risk groups rather than as universal screening tests [35].

### 2.7. High Cost

Biological tests' high costs have posed a significant hurdle in the process of estimating cardiovascular risk, particularly in low-income countries [50]. Considering the absence of specific prediction tools based on local epidemiological data in these countries, obtaining venous blood samples from every screened individual can be financially prohibitive. In response to this challenge, the World Health Organization (WHO) recently developed a prediction chart for cardiovascular risk (WHO/ISH charts) tailored for various global regions that relies on a nonlaboratory risk assessment procedure [51]. However, this model lacks validation in some countries [52].

## 3. Conclusion

Approximately half of all cardiovascular diseases (CVDs) stem from preventable risk factors, offering a promising avenue to curtail cardiovascular morbidity and mortality. Numerous cardiovascular risk scores are currently employed for predicting cardiovascular risk. However, many of these possess numerous limitations that can impact their effectiveness. Some scores lack validation in external cohorts, and others have demonstrated a tendency to miscalculate risk when applied to populations different from their origin relying on classical risk factors, which limit their sensitivity and do not explain all observed cardiovascular events. This is why it is recommended to prioritize the external validation of the existing models over the continuous development of new cardiovascular prediction tools in the face of this abundance. Lastly, as advancements continue in developing cardiovascular risk estimation tools, there is a clear need for the improvement of cardiovascular risk prediction tools that can be validated in various population, including a wide range of age and inexpensive variables. Currently, scientists are exploring the integration of new biomarkers, which hold promise in enhancing, although this potential improvement has yet to be substantiated.

## Figures and Tables

**Table 1:** The main identified cardiovascular risk scores.

CVR prediction model	Year	Origin	Cohort	Age	Risk factors
Framingham Heart Study [8]	1976	USA	5209	35-64	Age, sex, smoking, BP, TC, HDL-C
Framingham CHD [9]	1991	USA	5573	30-74	Age, sex, smoking, BP, TC, LHV, HDL-C, and DM
Framingham CVD [10]	1998	USA	5345	30-74	Age, sex, smoking, SBP, TC, HDL-C, DM
Frammingham-30 [11]	2008	USA	8491	30-74	Age, sex, smoking, SBP, TC, HDL-C, DM, BMI
REGICOR [20]	1978	Spain	15000	35-74	Age, sex, smoking, SBP, TC, LHV, HDL-C, DM
Dubbo study of the elderly [21]	1988	Australia	2805	Over 60	Age, sex, BMI, smoking, FH, SBP, AntiHyp, TC, HDL-C, DM, TRG, stress
PROCAM [12]	2002	Germany	5389	35-65	Sex, smoking, SBP, DM, HDL-C, LDL-C, TGC
SCORE [15]	2003	USA	205178	45-64	Age, sex, smoking, SBP, CT, HDL-C
ARIC [22]	2003	USA	14054	45-64	Sex, smoking, SBP, DM, HTA, HDL-C, LDL-C, TGC
Progetto CUORE [23]	2004	Italy	20647	35-69	Age, sex, smoking, HTA, SBP, TC, HDL-C, DM
PROCAM [12, 13]	2007	Germany	5389	35-65	Sex, smoking, SBP, HTA, TC, HDL-C, LDL-C, TGC
ASSIGN [14]	2007	UK	13297	30-74	Age, sex, smoking, SBP, HTA, TC, HDL-C, DM
Reynolds women [16]	2007	USA	16400	Over 45	Age, sex, smoking, SBP, TC, HDL-C
Reynolds men [17]	2008	USA	10724	50-80	Age, sex, smoking, SBP, TC, HDL-C
QRISK [18]	2007	UK	128317	35-74	Age, sex, smoking, HTA, SBP, DM, BMI
QRISK 2 [19]	2008	UK	1535583	35-74	Age, sex, smoking, HTA, SBP, DM, BMI
WHO/ISH charts [24]	1999	USA	14 regions	40-70	Age, sex, smoking, HTA, SBP, TC, DM
Pooled cohort equation ASCVD [25]	2013	USA	20843	40-79	Age, sex, smoking, SBP, HTA, TC, HDL-C, DM

SBP: systolic blood pressure; TC: total cholesterol; HDL-C: high-density lipoprotein cholesterol; BMI: body mass index; FH: family history; LVH: left ventricular hypertrophy; RVH: right ventricular hypertrophy; HTN: hypertension; AntiHyp: antihypertensives; DM: diabetes mellitus; TRG: triglycerides; USA: United States of America; UK: United Kingdom; WHO: World Health Organization.

## Data Availability

The data used to support the findings of this study are included within the article.

## Abbreviations

ASSIGN: Assessing Cardiovascular Risk to Scottish Intercollegiate Guidelines Network/SIGN to Assign Preventative Treatment
ASVCD: Atherosclerotic cardiovascular disease
CVD: Cardiovascular diseases
CVR: Cardiovascular risk
CVRF: Cardiovascular risk factor
BP: Blood pressure
CT: Cholesterol
FHS: Framingham Heart Study
NHEFS: The NHANES I Epidemiologic Follow-Up Study
WHO/ISH: World Health Organization/International Society of Hypertension.
